# Genome-wide analysis and characterization of *Dendrocalamus farinosus SUT* gene family reveal *DfSUT4* involvement in sucrose transportation in plants

**DOI:** 10.3389/fpls.2022.1118398

**Published:** 2023-01-20

**Authors:** Bin Deng, Xiaoyan Gu, Sen Chen, Meng Zhang, Suwei Hao, Lixian Wei, Ying Cao, Shanglian Hu

**Affiliations:** ^1^ Lab of Plant Cell Engineering, Southwest University of Science and Technology, Mianyang, Sichuan, China; ^2^ Engineering Research Center for Biomass Resource Utilizaiton and Modification of Sichuan Province, Mianyang, Sichuan, China

**Keywords:** *Dendrocalamus farinosus*, *SUT* gene, phytohormone responses, abiotic stress, genome-wide analysis, sucrose transportation, fiber formation

## Abstract

Sucrose is the main transported form of photosynthetic products. Sucrose transporter (SUT) participates in the translocation of sucrose from source to sink, which is important for the growth and development of plants. *Dendrocalamus farinosus* is an important economic crop in southwestern China because of its high growth rate, high fiber content, and dual usage for food and timber, but the mechanism of sucrose transportation in *D. farinosus* is unclear. In this study, a total of 12 *SUT* transporter genes were determined in *D. farinosus* by whole-genome identification. *DfSUT2*, *DfSUT7*, and *DfSUT11* were homologs of rice *OsSUT2*, while *DfSUT4* was a homolog of *OsSUT4*, and these four *DfSUT* genes were expressed in the leaf, internode, node, and bamboo shoots of *D. farinosus*. In addition, *DfSUT* family genes were involved in photosynthetic product distribution, ABA/MeJA responses, and drought resistance, especially *DfSUT4*. The function of *DfSUT4* was then verified in *Nicotiana tabacum*. DfSUT4 was localized mainly in the leaf mesophyll and stem phloem of *pDfSUT4::GUS* transgenic plant. The overexpression of *DfSUT4* gene in transgenic plant showed increases of photosynthetic rate, above-ground biomass, thousand grain weight, and cellulose content. Our findings altogether indicate that *DfSUT4* can be a candidate gene that can be involved in phloem sucrose transportation from the source leaves to the sink organs, phytohormone responses, abiotic stress, and fiber formation in plants, which is very important in the genetic improvement of *D. farinosus* and other crops.

## Introduction

Sucrose is the major long-distance-transported form of carbohydrate metabolites in plants (Deol et al., 2013; Wang et al., 2021). After having been synthesized by photosynthesis in mature leaves (source), sucrose is further transported to a variety of heterotrophic organs (sink) such as roots, developing leaves, flowers, and seeds (Barker et al., 2000; Sun et al., 2010). Sucrose transporters (SUTs; also called SUCs) are sucrose–H^+^ symporters that are localized in the vacuole membrane or plasma membrane (Zhu et al., 2015), and they are members of the major facilitator superfamily that contains 12 transmembrane-spanning helices (Aoki et al., 2012). In addition, the *SUT* family genes play key roles in sucrose loading and unloading from t source tissue to sink cells through the phloem to effect multiple processes of plant growth and development, such as phytohormone biosynthesis, abiotic stresses, photosynthesis, flower development, and fiber synthesis in many species (Srivastava et al., 2009; Slewinski and Braun, 2010; Payyavula et al., 2011; Wang et al., 2021). According to the direction and type of transmembrane transport of sucrose, SUT transporters in plants can be divided into three types: plasma membrane efflux carriers are active in the efflux of sucrose from the mesophyll cells to the apoplast, plasma membrane influx carriers function in the entry of sucrose from the apoplast into phloem cells, and tonoplast carriers are involved in the transport of sucrose between the vacuole and the cytoplasm (Ward et al., 1998; Lemoine, 2000).

To date, there are numerous studies that have shown SUTs as crucial not only for carbohydrate partitioning but also for responses to various phytohormones, abiotic stresses, and environmental factors in plants—for example, *Arabidopsis suc2* seedlings were smaller than the wild type in the absence of sucrose, and ABA treatment on *Arabidopsis suc2* mutant could increase the sucrose content in shoots but decrease the sucrose content in roots (Gottwald et al., 2000), suggesting that AtSUC2 is crucial for plant development and sucrose loading in *Arabidopsis* root (Gong et al., 2015). In contrast to other AtSUC transporters, AtSUC4 is localized in the tonoplast and participate in the translocation of sucrose from the vacuole into the cytoplasm (Weise et al., 2000). Consistently with *SUC4* from *Arabidopsis*, *PtaSUT4* from poplar and *OsSUT2* from rice are also localized to the tonoplast and mediate sucrose efflux from the source leaves (Eom et al., 2011). Moreover, PtaSUT4-repressed poplar showed increased leaf-to-stem biomass ratio and sucrose content in the source leaves because of the translocation of sucrose to the sink organs which was inhibited in transgenic plants (Payyavula et al., 2011). The plasma membrane-localized *OsSUT4* functions in the apoplastic phloem loading of sucrose. The rice *sut4* mutant showed a dwarf phenotype, and the yield of rice was decreased (Chung et al., 2014).

In addition, *AtSUC2*, *AtSUC4*, and *AtSUC9* are all associated with ABA treatment and drought/cold stress (Gong et al., 2015; Jia et al., 2015). *OsSUT2* promotes photosynthesis and sucrose distribution in plants under drought and salt stress (Ibraheem et al., 2011). *PtaSUT4* transporters also function in photosynthesis (Frost et al., 2012) and drought response (Harding et al., 2020). Furthermore, *PaSUT* and *OsSUT1* transporters are associated with phytohormone (auxin and cytokinin) signaling pathways in the translocation of sucrose in *Petunia axillaris* flower (Rolland et al., 2002; Iftikhar et al., 2020) and rice seeds (Zhao et al., 2022), respectively. More importantly, *PttSUT3* transporters are responsible for carbon delivery and formation of wood fibers in a hybrid aspen (Mahboubi et al., 2013).

Bamboo is widely scattered in China, and products made of bamboo have been utilized by humans since the ancient times (Qi et al., 2015). *D. farinosus* is one of the essential economic bamboo species in southwestern China, which has many advantages including high growth rate and disease resistance, cold and drought tolerance, and dual usage for food and timber (Zakikhani et al., 2014; Chen et al., 2021; Pei et al., 2022). In particular, *D. farinosus* is also an excellent paper pulp bamboo species because of its high fiber content, stiffness, and low density (Xiong et al., 2012; Imran et al., 2022). Sucrose is the predominant form of transported carbon, which is necessary to synthesize fiber (Rennie and Turgeon, 2009), and *SUT* transporter genes are crucial to carbon delivery and fiber formation in some plants (Zhang et al., 2017; Yadav et al., 2022). However, the mechanisms of sucrose translocation between the source leaves and the sink organs of *D. farinosus* are unclear. In this study, we reported the phylogenetic analysis and the whole-genome identification of *SUT* genes as well as their chromosomal localization and expression pattern in *D. farinosus*. Most *DfSUT* genes were involved in ABA/MeJA signaling pathways and drought resistance. We then verified the transport function of *D. farinosus DfSUT4* as a rice *OsSUT4* homolog in transgenic *Nicotiana tabacum*. Our results indicated that *DfSUT4* is a candidate gene in carbohydrate transport and improving the photosynthetic rate as well as involved in the phloem transportation of sucrose from the source leaves to the sink organs, phytohormone responses, abiotic stress, and fiber formation in *D. farinosus* and other crops.

## Results

### Identification of *SUT* genes in *D. farinosus*

The identified *SUT* sequences in rice were searched for the corresponding *SUT* homologous gene in *D. farinosus*. According to the chromosomal location, we identified 12 genes of the *DfSUT* transporter family (**Table 1**), namely *DfSUT1*–*DfSUT12*. Detailed information about the 12 *DfSUT* genes, including the predicted length of CDSs, encoded proteins, and physicochemical parameters, are presented in **Table 1**. The length of *DfSUT* CDSs ranged from 291 to 1,803 bp, with an average length of 1,481 bp. The protein size of *DfSUT* was an average of 493 aa, which ranged from 96 to 600 aa. The molecular weight ranged from 10.54 kDa (*DfSUT12*) to 64.06 kDa (*DfSUT9*). The pI for all *DfSUT* genes was below 10.0, with an average value of 8.1; only one gene product (*DfSUT1*) was below 5.0. *DfSUT* genes were predicted to localize to the plasma membrane or tonoplast. Among them, *DfSUT1* was not assembled on the chromosome of Liang Shan Cichlid, so it was not discussed in the following analysis.

**Table 1:** Detailed information about the 12 predicted *DfSUT* genes in *D. farinosus*.

Locus name	Gene name	Clade	Os orthologs	pI	Molecular weight (Da)	Open reading frame length (bp)	Protein length (aa)	Location	Subcellular localization^a^
Dfa0G064610.1	*DfSUT1*	III	OsSUT3	4.77	59,552.72	1,683	560	Contig01867	Plasma membrane
DfaA10G000160.1	*DfSUT2*	II	OsSUT2	8.96	53,294.26	1,500	499	DfA01	Tonoplast
DfaB03G013150.1	*DfSUT3*	III	OsSUT5	8.66	53,560.78	1,500	499	DfB03	Plasma membrane
DfaB03G024800.1	*DfSUT4*	III	OsSUT4	6.18	63,938.51	1,803	600	DfB03	Plasma membrane
DfaB08G004380.1	*DfSUT5*	III	OsSUT1	8.84	55,356.67	1,575	524	DfB08	Plasma membrane
DfaB09G006030.1	*DfSUT6*	III	OsSUT3	8.32	51,342.23	1,464	487	DfB09	Plasma membrane
DfaB10G000010.1	*DfSUT7*	II	OsSUT2	9.28	53,431.54	1,503	500	DfB10	Tonoplast
DfaC03G011900.1	*DfSUT8*	III	OsSUT5	8.99	55,931.41	1,578	525	DfC03	Plasma membrane
DfaC03G022010.1	*DfSUT9*	III	OsSUT4	6.63	64,062.84	1,800	599	DfC03	Plasma membrane
DfaC08G007580.1	*DfSUT10*	III	OsSUT1	8.95	54,922.15	1,566	521	DfC08	Plasma membrane
DfaC08G021670.1	*DfSUT11*	II	OsSUT2	9.19	53,556.58	1,512	503	DfC08	Tonoplast
DfaC11G004310.1	*DfSUT12*	III	OsSUT4	8.77	10,535.14	291	96	DfC11	Plasma membrane

a Subcellular localization of *D. farinosus* sucrose transporters (SUTs) based on the subcellular localization of rice homologous SUTs (Hu et al., 2021).

### Analysis of the phylogenetic relationships, chromosomal localization, and gene structure of *SUT* families in *D. farinosus*

**Figure 1:**
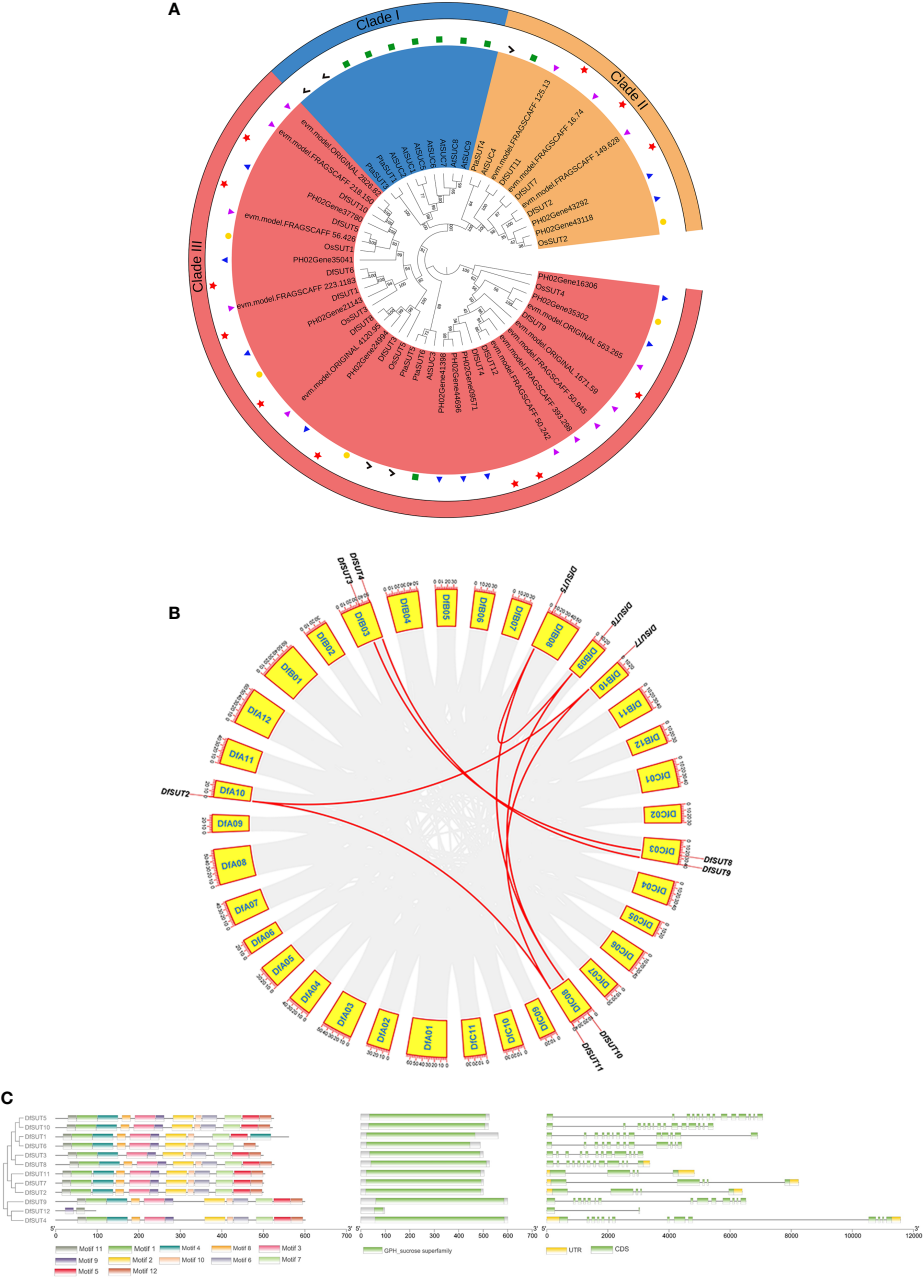
Analysis of the phylogenetic evolutionary, chromosomal localization and gene structure of the *SUT* family in *D. farinosus.* **(A)** Phylogenetic tree of the *SUT* family in *D. farinosus.* Based on the amino acid sequences of *D. farinosus*, *Phyllostachys edulis*, *D. latiflorus munro*, *Arabidopsis*, rice, and *Populus* L., phylogenetic trees were generated by the neighbor joining method using MEGA 7.0.21. The *SUT* members are divided into three branches: clades I, II, and III. The species are labeled with different colors: Df, *D. farinosus* (red); *Phyllostachys edulis* (blue); *D. latiflorus munro* (purple); At, *Arabidopsis* (green); Os, rice (yellow); Pta, *Populus* L. (black). **(B)** Chromosomal localization and gene duplication analysis of *DfSUT* genes in the genome of *D. farinosus.* The *DfSUT* genes are localized on different chromosomes. Chromosome numbers are indicated in the yellow box. The numbers on the chromosome boxes represent the sequence length in megabases. Gene pairs with sibling relationships are connected by a red line. **(C)** Conserved motif and gene structure analysis of the *SUT* family in *D. farinosus*. The three maps of conserved motif analysis, conserved structural domains, and exon/intron structure of *SUT* genes were merged in the order of phylogenetic tree by the Gene Structure View online software analysis of TBtools software.

To comprehensively analyze the evolutionary relationships between SUT families in *D. farinosus*, *Phyllostachys edulis*, *D. latiflorus munro*, *Arabidopsis thaliana*, rice, and *Populus* L., we constructed a neighbor-joining (NJ) phylogenetic tree of *SUTs* based on 55 full-length SUT protein sequences, including 12 sequences from *D. farinosus*, 10 sequences from *Phyllostachys edulis*, 13 sequences from *D. latiflorus munro*, nine sequences from *Arabidopsis thaliana*, five sequences from rice, and six sequences from *Populus* L. (**Figure 1A**). All three woody bamboos contain more *SUT* genes than *Arabidopsis thaliana*, rice, and *Populus* L. According to the phylogenetic analysis, plant SUTs are divided into three types. Type I and type II SUTs are localized to the plasma membrane, and type III SUTs are localized to the vacuolar membrane (Reinders et al., 2012). The phylogenetic tree showed that the 55 SUT proteins could be divided into three distinct groups (clade I–clade III; **Figure 1A**). All 12 *SUT* genes in *D. farinosus* could be divided into two clades (clade II and clade III), such as three *DfSUTs* (*DfSUT2*, *DfSUT7*, and *DfSUT11*) that belonged to clade II. The other nine *DfSUTs* belonged to clade III. However, none of the *SUTs* in all three woody bamboos and rice belonged to clade I. *DfSUT2*, *DfSUT7*, and *DfSUT11* were homologs of rice *OsSUT2*, *Arabidopsis AtSUC4*, and *Populus* L. *PtaSUT4*. While *DfSUT4*, *DfSUT9*, and *DfSUT12* were homologs of *AtSUC3*, *OsSUT4*, *PtaSUT5*, and *PtaSUT6*, suggesting that they might have a similar function. Interestingly, we found that all three woody bamboos contained almost an equal number of *SUT* genes, and they all had more *SUT* genes than the other species.

The chromosomal distribution showed that 10 of the 12 *DfSUT* genes were mapped on seven chromosomes in *D. farinosus* (**Figure 1B**) (**Supplementary Table S4**). The result showed that four chromosomes (DfA10, DfB08, DfB09, and DfB10) harbored only one gene, and they were *DfSUT2*, *DfSUT5*, *DfSUT6*, and *DfSUT7*, respectively. Three chromosomes (DfB03, DfC03, and DfC08) contained two genes, such that chromosome DfB03 contained *DfSUT3* and *DfSUT4*, chromosome DfC03 contained *DfSUT8* and *DfSUT9*, and chromosome DfC08 contained *DfSUT10* and *DfSUT11*. In addition, among the 12 *DfSUT* family genes, there were 10 genes with existing gene duplication. Seven gene pairs connected with each other, representing fragment duplication, such as *DfSUT2* and *DfSUT7*, *DfSUT2* and *DfSUT11*, *DfSUT7* and *DfSUT11*, *DfSUT3* and *DfSUT8*, *DfSUT4* and *DfSUT9*, *DfSUT5* and *DfSUT6*, *DfSUT5* and *DfSUT10* (**Figure 1B**). In addition, we estimated the approximate dates of *DfSUT* gene duplication events and the gene evolutionary selection pressure by measuring the Ka and Ks values and the Ka/Ks ratios (**Supplementary Table S5**). The Ka/Ks ratios of the *DfSUT* genes were all less than 1, indicating that they were all in a purifying selection condition.

We then analyzed the conserved motif and gene structure of the *SUT* family in *D. farinosus* (**Figure 1C**). The amino acid sequences of DfSUT in **Table 1** were used to construct the phylogenetic tree separately. The conserved motifs and structural domains of the family were identified using MEME and GSDS online tools, respectively. The structural analysis maps of the above-mentioned three sequences were combined and plotted using TBtools (**Figure 1C**). The result showed that all DfSUT proteins contain 11 or 12 motifs and have similar conserved structural domains, except for DfSUT12 which has only two motifs. In the *SUT* family, eight *SUT* genes contain 14 exons and 13 introns, while three *SUT* genes contain five exons and four introns. Interestingly, we found that *DfSUT12* only has two exons and one intron, and seven *SUT* genes including *DfSUT12* have no upstream and downstream untranslated regions (**Figure 1C**).

### Analysis of the expression patterns of *SUT* genes and C content in different developmental phases/tissues in *D. farinosus*

**Figure 2:**
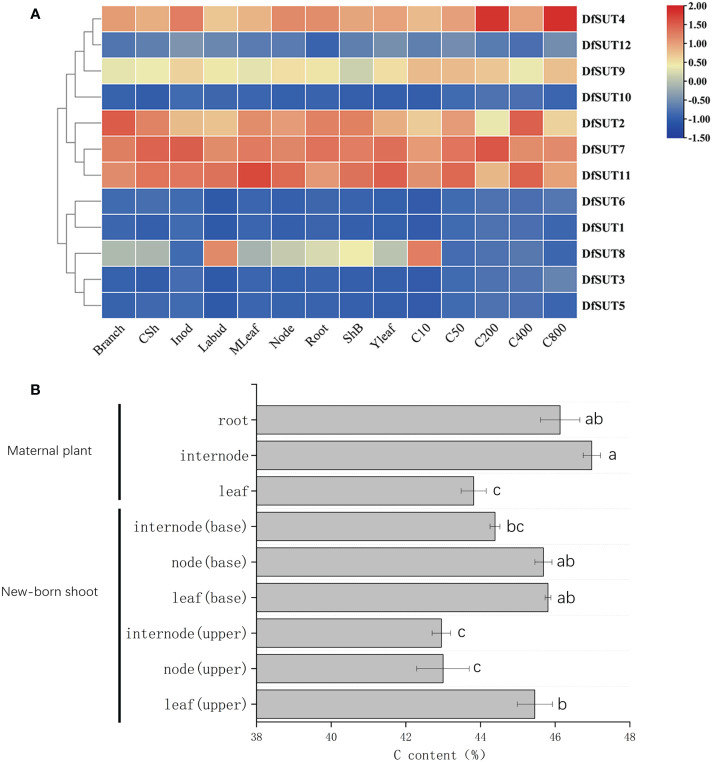
Analysis of transcriptome expression profiles of *SUT* and C content in different developmental phases/tissues of *D. farinosus.* **(A)** Transcriptome expression profiles of *SUT* at different developmental stages and various parts in *D. farinosus*. The red and blue colors correspond to the strong and weak expression of the genes, respectively. **(B)** C content in different developmental phases and tissues in *D. farinosus.* Data are detected as the mean ± SD from three independent experiments. The *P*-values of Student’s test for different developmental phases and tissues in *D. farinosus* are denoted with letters (*P *< 0.05).

To determine the dynamic gene expression of *SUT* genes in *D. farinosus*, we performed RNA-Seq analysis of 12 *SUT* protein-coding genes in 14 organs and developmental stages of *D. farinosus*, including branch, culm sheath (CSh), internode (Inod), lateral bud (Labud), mature leaf (MLeaf), node, root, sheath of bamboo shoot (ShB), young leaf (Yleaf), 10-cm shoot (C10), 50-cm shoot (C50), 200-cm shoot (C200), 400-cm shoot (C400), and 800-cm shoot (C800). According to the transcriptome heat map, *DfSUT2*, *DfSUT4*, *DfSUT7*, and *DfSUT11* showed a wide range of expression levels and distinct regulation during *D. farinosus* development (**Figure 2A**). These four *SUT*-encoding genes (*DfSUT2*, *DfSUT4*, *DfSUT7*, and *DfSUT11*) were abundantly expressed in the branch, leaf, and root (**Figure 2A**), suggesting that they were associated with the transportation of sucrose from the source leaf to the sink organs. They were all highly expressed in bamboo shoots (**Figure 2A**), such as *DfSUT2* and *DfSUT11* in 400-cm-tall shoot, *DfSUT4* in 200-cm- and 800-cm-tall shoots, and *DfSUT7* in 200-cm-tall shoot, suggesting that they were all associated with the rapid growth of shoots. Interestingly, *DfSUT4*, *DfSUT7*, and *DfSUT11* were abundantly expressed in internode and node, suggesting that they were involved in sucrose accumulation in the node and internode of *D. farinosus*. *DfSUT8* was only highly expressed in the lateral bud and 10-cm shoot, suggesting that it is important for the development of buds and shoots.

We then measured the C content in different developmental phases and tissues in *D. farinosus* (**Figure 2B**). Consistent with the expression patterns of *SUT* genes, the high C content in the mature internode suggested that carbohydrate transportation by DfSUT proteins was necessary to synthesize cellulose in the internode of *D. farinosus*. More importantly, the new leaf (base), new node (base), and mature root of *D. farinosus* contained the same high C content (**Figure 2B**). These results showed that, except the leaf source and the root sink, the bamboo node that consists of complex vascular bundles in *D. farinosus* might be a temporary sink for the accumulation of sucrose for some specific functions.

### Prediction of cis-regulatory elements in the *SUT* families of *D. farinosus*

**Figure 3:**
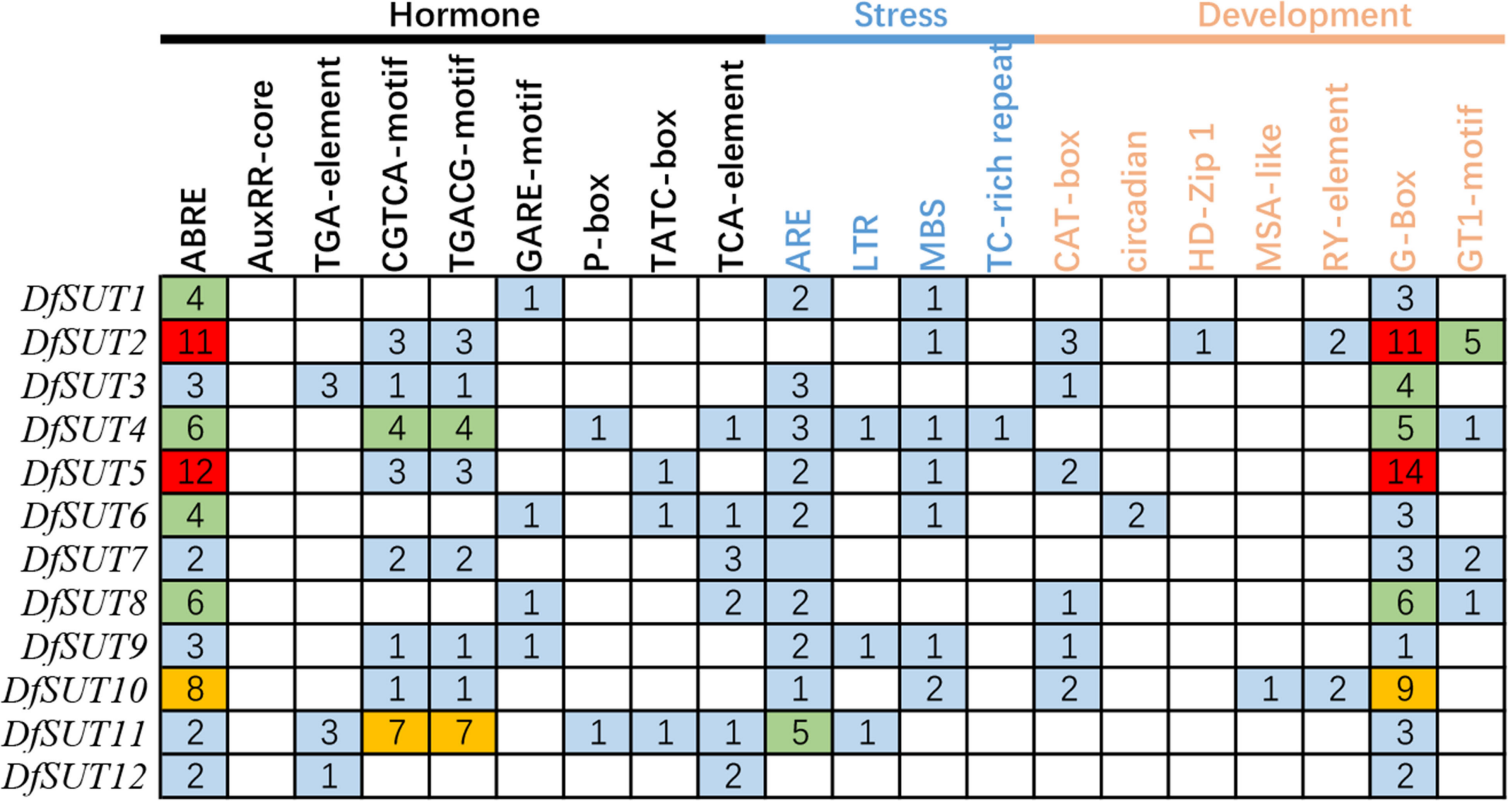
The *cis*-regulatory elements involved in phytohormone, development, and stress responses in the upstream regions of *DfSUT* gene promoters. ABRE, abscisic acid-responsive element; AuxRR core and TGA element, auxin-responsive element; CGTCA-motif and TGACG-motif, MeJA-responsive elements; GARE-motif, P-box and TATC-box, gibberellin-responsive elements; TCA element, salicylic acid-responsive elements; ARE, involved in anaerobic induction; LTR, low temperature-responsive element; MBS, TC-rich repeats, involved in defense and stress response; CAT box, circadian, HD-Zip I, MSA-like, and RY-element involved in meristem expression, circadian control differentiation of the palisade mesophyll, cell cycle regulation, and seed-specific regulation, respectively; G-box, GT1-motif, light-responsive elements.

To investigate the responses to various factors by *DfSUT* members, the promoter (2 kb upstream) of these genes was submitted to the PlantCARE server to predict its promoter cis-regulatory elements. We identified 20 cis-regulatory elements in *DfSUT* gene promoter, such as phytohormone response elements (abscisic acid, auxin, methyl jasmonate, gibberellin, and salicylic acid), abiotic stress response elements (anaerobic, low-temperature, drought, defense, wound, and light), and growth and development response elements (meristem expression, circadian control, mesophyll cells differentiation, cell cycle regulation, and seed-specific regulation) (**Figure 3**). The result showed that most *DfSUT* genes were associated with plant hormones, stress, growth, and development, suggesting that *DfSUT* genes are involved in multiple physiological processes through a variety of environmental adaptations. In addition, ABRE-motif, CGTCA-motif, TGACG-motif, MBS-motif, and G-Box-motif were most enriched in the *DfSUT* promoter regions (**Figure 3**), which indicated that most *DfSUT* genes might be involved in the responses of ABA, MeJA, drought, and light—for example, six *DfSUT* genes (*DfSUT2*, *DfSUT4, DfSUT5*, *DfSUT6*, *DfSUT8*, and *DfSUT10*) were related to the responses of ABA, and five *DfSUT* genes (*DfSUT2*, *DfSUT4*, *DfSUT5*, *DfSUT7*, and *DfSUT11*) were related to the responses of MeJA. Moreover, *DfSUT2* and *DfSUT5* included more than 10 motifs in the promoter that were associated with ABA and light, respectively. *DfSUT4* contained about five motifs in the promoter that were associated with ABA, MeJA, and light, respectively.

### Analysis of the photosynthetic rate and response to phytohormone treatment and drought stress of the *SUT* family in *D. farinosus*

**Figure 4:**
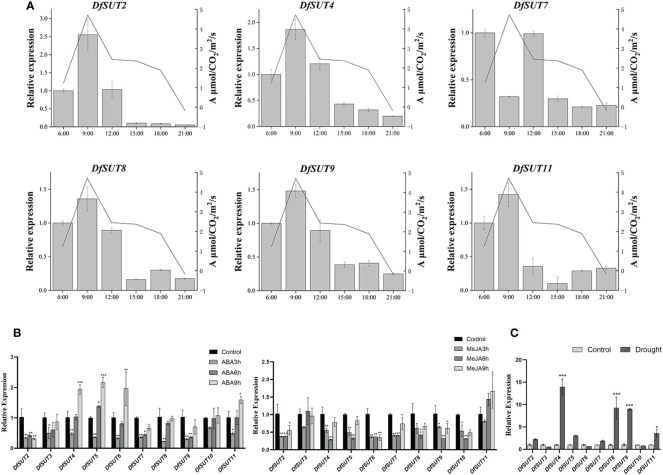
Analysis of photosynthetic rate and response to different treatments of the *SUT* family in *D. farinosus*. **(A)** Relative expression of *DfSUT* at different times of the day and the net photosynthetic reaction rate at the corresponding time periods. Total RNA was extracted from *D. farinosus* leaves every 3 h (from 6 a.m. to 9 p.m.) during the day. **(B)** Relative expression of *DfSUT* in response to 100 mM ABA treatment and 100 mM MeJA treatment for 3, 6, and 9 h, respectively. **(C)** Relative expression of *DfSUT* under drought treatment. *, P < 0.05, **, P < 0.01, ***, P < 0.001, Student's t-test.

To determine the transport of photosynthetic products by SUT proteins, we measured the transcript levels and net photosynthetic reaction rate in *D. farinosus* leaves every 3 h (from 6 a.m. to 9 p.m.). The results showed that the expression of *DfSUT* remained basically consistent with the change in the net photosynthetic reaction rate, except *DfSUT7* (**Figure 4A**). The transcript levels of five *DfSUT* genes (*DfSUT2*, *DfSUT4*, *DfSUT8*, *DfSUT9*, and *DfSUT11*) increased with the increase of the net photosynthetic reaction rate in *D. farinosus*, and both of them reached the highest levels at 9 a.m. These results indicated that the expression of these *DfSUT* genes was involved in the transport of photosynthetic products.

More importantly, we determined the response of *DfSUT* genes to phytohormone treatment (ABA and MeJA) and drought stress. Consistent with the analysis of the cis-regulatory elements of the *DfSUT* gene promoter, the results showed that the expression of nine *DfSUT* genes decreased within 3 h and then increased within 9 h under the treatment of exogenous ABA, except *DfSUT2*. Compared with the control that was untreated, the expression of *DfSUT4*, *DfSUT5*, and *DfSUT6* was much higher in response to ABA within 9 h, but the transcript levels of *DfSUT2* and *DfSUT7* showed significant declines within 9 h in response to ABA (**Figure 4B**). However, the transcript levels of six *DfSUT* genes (*DfSUT2*, *DfSUT3*, *DfSUT4*, *DfSUT5*, *DfSUT7*, and *DfSUT11*) decreased within 3 h and then showed a rebound within 9 h in response to MeJA. Compared with the control that was untreated, the transcript levels of *DfSUT2*, *DfSUT6*, and *DfSUT10* showed significant declines, but the expression of *DfSUT11* showed an increase within 9 h in response to MeJA (**Figure 4B**). Moreover, the transcript levels of *DfSUT4*, *DfSUT8*, *DfSUT9*, and *DfSUT11* showed significant increases in response to the drought treatment (**Figure 4C**).

### Overexpression of *DfSUT4* gene in *N. tabacum* showed increases of photosynthesis, biomass, and cellulose content

**Figure 5:**
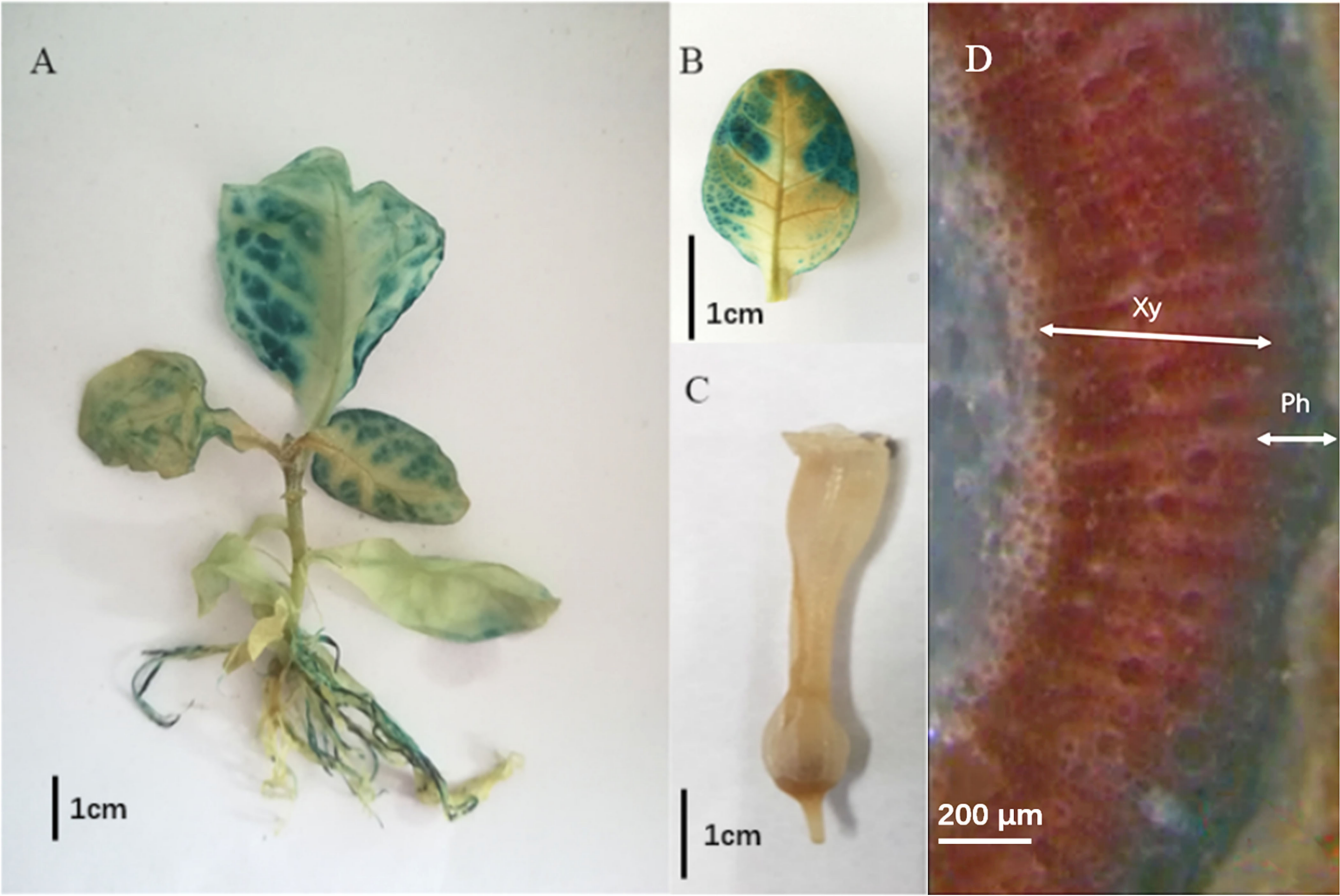
Expression of *pDfSUT4::GUS* in *N. tabacum*. **(A)** Intact plant. **(B)** Leaf. **(C)** Flower. **(D)** Stem section. Xy, xylem, Ph, phloem.

Since we have found that the *DfSUT4* gene was involved in the photosynthetic product transport and the responses to various phytohormone treatment (ABA and MeJA) and drought stress, we then verified the transport function of *DfSUT4* in transgenic *N. tabacum* lines. The result showed that GUS activity was mainly detected in the mesophyll of leaves, primary roots, lateral roots, and root hair in *pDfSUT4::GUS* transgenic plant, but not in flower organ (**Figures 5A–C**). The *DfSUT4* gene was also found to be expressed in the phloem of the stem section in *N. tabacum* (**Figure 5D**).

**Figure 6:**
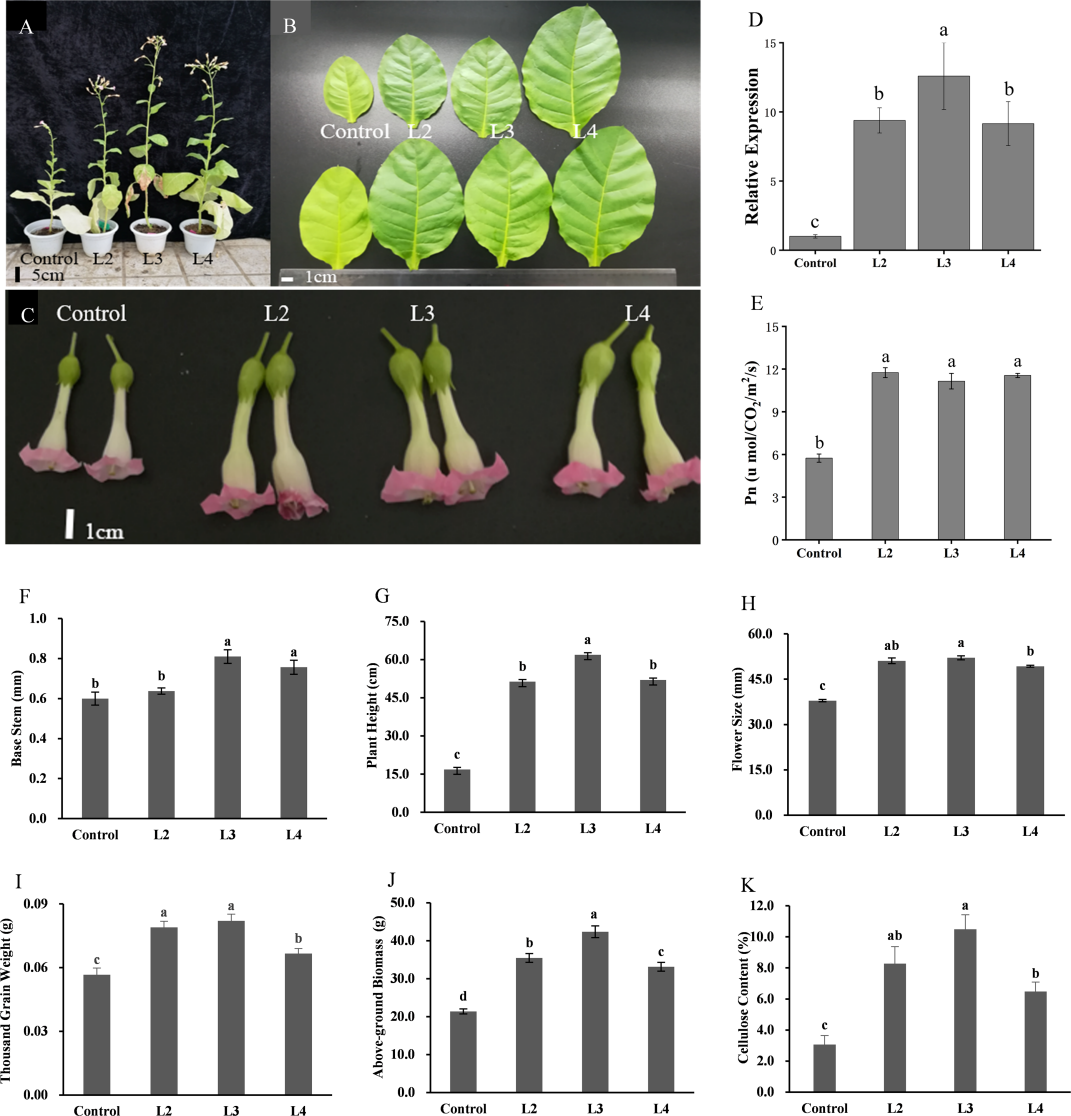
Morphological phenotypes, statistics, and above-ground biomass/cellulose content of control and three transgenic *N. tabacum* lines. **(A)** 3-month-old plants. **(B)** Leaf blades of 2-month-old plants. **(C)** Phenotype of the flower. **(D)** Relative expression of *DfSUT4* in transgenic *N. tabacum*. **(E)** Net photosynthetic reaction rate of control and transgenic lines. **(F–I)** Morphologic index of control and transgenic lines. **(J, K)** Above-ground biomass and cellulose content of control and transgenic lines. Data are expressed as the mean ± SD from six independent experiments. The *P*-values of Student’s test for control and transgenic lines are denoted with letters (*P* < 0. 05).

Moreover, the result showed that, compared with CK, *DfSUT4* transgenic *N. tabacum* showed phenotypes of taller stem and noticeably larger leaves, flowers, and fruits (**Figures 6A–D, F–I**). The net photosynthetic reaction rate increased nearly twofold in transgenic *N. tabacum* (**Figure 6E**), suggesting that *DfSUT4* genes might be involved in the distribution of carbohydrate from the source leaves. Carbohydrates can be synthesized by photosynthesis in mature leaves and then transported to other sink tissues (Barker et al., 2000; Sun et al., 2010). To explore the distribution mechanism of the increased photosynthetic products, we measured the above-ground biomass and cellulose content of stem in three transgenic lines. The results showed that, compared with the control line, the above-ground biomass in transgenic *N. tabacum* line 3 has increased nearly twofold compared with the control lines (**Figure 6J**), and the cellulose content of the stem in line 3 was increased by more than threefold (**Figure 6K**). Therefore, these results suggested that *DfSUT4* is important for the translocation of sucrose from the leaf to the sink organs through the phloem and promotes cellulose synthesis in plants.

## Discussion

In higher plants, sucrose is produced by leaf photosynthesis and then metabolized into hexoses (glucose and fructose), which are necessary to synthesize cellulose, proteins, and starch and generate energy in the sink tissues (Ruan, 2014). The SUT proteins play key roles in the translocation of sucrose from the source to the sink through the phloem to support plant growth and development (Slewinski and Braun, 2010; Deol et al., 2013). *D. farinosus* is an ideal economic crop in southwestern China because of its high fiber content and dual usage for food and paper pulp (Chen et al., 2021; Imran et al., 2022; Pei et al., 2022). Sucrose is the main substrate of fiber synthesis, and studying the function of *SUT* genes in regulating sucrose transportation helps to illustrate the synthesis mechanism of fiber in plants (Yadav et al., 2022)—for example, *GhSUT1* and *GhSUT3* are crucial to regulate sucrose accumulation in cotton fibers (Zhang et al., 2017; Yadav et al., 2022). *PttSUT3* transporters are responsible for the synthesis of wood fibers in a hybrid aspen (Mahboubi et al., 2013).

Herein we identified 12 main *SUT* transporter genes in *D. farinosus*, which were distributed in eight chromosomes in groups A, B, and C of *D. farinosus*, respectively (**Table 1**; **Figure 1B**). We analyzed the phylogenetic relationships of *SUT* genes from three woody bamboos including *D. farinosus*, *Phyllostachys edulis*, and *D. latiflorus munro* and found that some *SUT* genes from *Phyllostachys edulis* and *D. latiflorus munro* clustered together, respectively. However, the *SUT* genes from *D. farinosus* were relatively scattered (**Figure 1A**). All three woody bamboos contained more *SUT* genes than *Arabidopsis* and rice, suggesting that *SUT* genes were vital to synthesize fiber with high quality and content in woody bamboos. The phylogenetic analysis also showed that *DfSUT2*, *DfSUT7*, and *DfSUT11* were homologs of *OsSUT2* and *PtaSUT4*, and *DfSUT4* is a homolog of *OsSUT4* (**Figure 1A**); all of these four *DfSUT* genes were mainly expressed in the leaves of *D. farinosus* (**Figure 2A**). These results suggested that *DfSUT2*, *DfSUT7*, and *DfSUT11* might be important for sucrose efflux from the vacuole to the apoplast in the source leaves of *D. farinosus* (Eom et al., 2011), and *DfSUT4* might function in the apoplastic loading of sucrose from the leaf mesophyll to the phloem (Chung et al., 2014). Chen et al. (2022) revealed that the bamboo node consists of sophisticated porous vascular bundles, microfibers, and twist-aligned nanofibers, and they are essential to the structural stability maintenance and body growth of bamboo. We found that *DfSUT4*, *DfSUT7*, and *DfSUT11* were highly expressed in both the internode and node of *D. farinosus* (**Figure 2A**). The same high C content was detected in the new leaf (base), new node (base), and mature root in *D. farinosus* (**Figure 2B**), suggesting that the directional channels, including multiscale sieve tubes and vessels in the node, might serve as a temporary sink for the accumulation of sucrose for some specific functions, such as supply of nutrients for the rapid growth of bamboo at night, and these *DfSUT* genes were also involved in fiber formation in the internode of *D. farinosus*.

The involvement of sucrose transporters in phytohormone signaling and abiotic responses was already described in other species—for example, SlSUT2 and StSUT4 proteins were found to interact with brassinosteroid signaling, ethylene sensing, gibberellic acid responses, and auxin transport in tomato and potato (Bitterlich et al., 2014; Garg et al., 2022a). PaSUT and OsSUT1 were found to interact with hormone (auxin and cytokinin) signaling pathways to regulate the carbohydrate partitioning in *Petunia axillaris* flower (Rolland et al., 2002; Iftikhar et al., 2020) and the reproductive organ of rice (Zhao et al., 2022), respectively. Consistent with these conclusions, we found that *DfSUT4*, *DfSUT5*, and *DfSUT6* showed significant increases in expression in response to ABA treatment within 9 h (**Figure 4B**). *DfSUT2*, *DfSUT6*, and *DfSUT10* showed significant decreases in response to MeJA treatment within 9 h (**Figure 4B**). The drought treatment also increased the transcript levels of *DfSUT4*, *DfSUT8*, *DfSUT9*, and *DfSUT11* (**Figure 4C**), so these results showed that the *DfSUT* family genes interacted with ABA and MeJA signaling pathways and drought resistance, which helped the plants to be more adaptable to environmental changes, especially the *DfSUT4* gene (**Figure 4**). We also found that the *DfSUT* genes were resistant to *Puccinia striiformis* (not shown).

Then, we verified the transport function of *DfSUT4* in *N. tabacum.* The OsSUT4 transporters were localized in the plasma membrane (PM), functioned in apoplastic phloem loading of sucrose, and increased the rice yield (Chung et al., 2014). Consistently, we found that *DfSUT4* was localized in the plasma membrane (not shown), and the GUS activity was expressed in the mesophyll, roots, and stem phloem of *pDfSUT4::GUS* transgenic plant (**Figure 5**). The overexpression of the *DfSUT4* gene in transgenic plant showed increases of photosynthesis capacity, above-ground biomass, thousand grain weight, and cellulose content and promoted the development of flower and seed in transgenic plant (**Figure 6**). These results suggested that *DfSUT4* can be a candidate gene to regulate the phloem transportation of sucrose from the source leaves to the sink organs, phytohormone responses, abiotic stress, and fiber formation in the internode of *D. farinosus*.

Partially functional redundancy existed in nine different *Arabidopsis AtSUC* genes (Garg et al., 2022b), such as the PM-localized AtSUC1 protein which interacted with the vacuolar AtSUC4 protein to mediate sucrose allocation in plants (Schulze et al., 2003; Kruegel et al., 2012). Recent studies revealed that SUT proteins are dynamic within cells, and SNARE protein is important to the subcellular movement of SUT transporters (Garg et al., 2020). At present, the interacting proteins of DfSUT, the functional redundancy between different DfSUT proteins, and the mechanisms of the *DfSUT4* genes involved in phytohormone responses, abiotic stress, and fiber formation in *D. farinosus* need to be analyzed further.

## Materials and methods

### Identification of the *SUT* genes of *D. farinosus*

The *SUT* family genes of rice were queried using the Uniprot database (https://www.uniprot.org/), and the candidate *SUT* sequences were obtained from the whole genomic data of *D. farinosus* (https://www.ncbi.nlm.nih.gov/, PRJNA923443) using HMMER 3.0 (*E* ≤ 10–20). The Conserved Domain Search (https://www.ncbi.nlm.nih.gov/Structure/cdd/wrpsb.cgi) online software in NCBl was used to remove the sequences that did not include the GPH sucrose superfamily domain. There were 12 *SUT* family members annotated in *D. farinosus* for further study (**Table 1**).

### Phylogenetic analysis of the *SUT* family of *D. farinosus*

The SUT family protein sequences of *Arabidopsis thaliana*, rice, *Populus* L., *Phyllostachys edulis*, and *Dendrocalamus latiflorus munro* were downloaded from UniProt (http://www.uniprot.org) (**Supplementary Table S1**). By using the Clustal W program in MEGA 6.06 software, we performed a multiple sequence alignment analysis of the SUT protein between *D. farinosus* and other species and constructed a phylogenetic tree by using the NJ method using MEGA 7.0.21 with bootstrap option *n* = 1,000 (Chen et al., 2020).

### Chromosomal localization and syntenic analysis of *DfSUT* genes

The Multiple Collinearity Scan toolkit (MCScanX) was used to analyze the internal synteny relationship and obtain syntenic gene pairs in the *SUT* family genes of *D. farinosus* (Wang et al., 2012). The syntenic analysis maps were then constructed using the Dual Systeny Plotter software (https://github.com/CJ-Chen/TBtools) (Liu et al., 2017).

### Gene structure and conserved motif analysis of the *SUT* family of *D. farinosus*

The conserved motifs of *DfSUTs* were analyzed using the MEME online tool (version 5.3.0, http://meme-suite.org/tools/meme) (Bailey et al., 2015), and the maximum motif search value was set at 10. Motif analysis and gene structure visualization were performed *via* TBtools. The Gene Structure Display Server 2.0 online tool (http://gsds.gao-lab.org/index.php) (Hu et al., 2015) was used to display the structure of *DfSUT* genes and the genomic length and organization of introns/exons. The 2,000-bp upstream promoter sequences of *DfSUT* genes were uploaded to the PlantCARE database (Lescot et al., 2002) (http://bioinformatics.psb.ugent.be/webtools/plantcare/html/), and the cis-elements were subsequently screened manually.

### Carbon content measurement

One-year-old *D. farinosus* materials were potted in a growing chamber wherein the condition was kept at 16-h light/8-h dark, and the temperature was 23–25°C. The samples from maternal plants and newborn shoots which included root, internode, leaf, and node were collected for the analysis of C content. All samples were de-enzymed at 100°C, dried at 85°C to a constant weight, and then ground. The total C content was measured using the potassium dichromate–sulfuric acid oxidation method (Cai et al., 2011). The experiment was repeated three times.

### Plant materials and hormone treatment

All materials used in this study were grown in a greenhouse at the Institute of Bamboo Research, Southwest University of Science and Technology. The leaves of 1-year-old *D. farinosus* were collected every 3 h (from 6:00 a.m. to 9:00 p.m.) during the day for the measurement of net photosynthetic reaction rate by using LCpro-SD photosynthesis equipment, and six independent biological replicates were performed. In addition, the young leaves of 1-year-old *D. farinosus* were sprayed with 100 mM ABA and MeJA, respectively, and the leaf samples were taken before treatment and after 3, 6, and 9 h of treatment.

### RNA extraction and qRT-PCR

Total RNA was extracted from *D. farinosus* leaves by using TRIzol reagent (Tiangen, Beijing, China), and DNase I was used to purify potentially genomic DNA. The quality of total RNA was checked by 1% denaturing agarose gel and a NanoDrop 2000 spectrophotometer (Thermo Fisher Scientific, Beijing, China). First-strand cDNA synthesis was performed by using the FastKing cDNA First Strand Synthesis Kit (Tiangen, Beijing, China). Specific primers were designed by using Primer Premier 5.0 (**Supplementary Table S2**). The internal reference gene was *TUBLIN*. The transcript levels of *DfSUT* were analyzed by real-time quantitative PCR assay using SYBR qPCR Master MIX kit (Vazyme, Nanjing, China) and CFX96TM Real-Time System thermal cycler (Bio-Rad, CA, USA). The relative expression levels of the *SUT* gene were calculated by using the 2^-ΔΔCt^ method (Livak and Schmittgen, 2001). The analysis included three biological replicates, and each had three technical replicates. The expression levels under different treatments were shown by histograms using the mean values.

### RNA sequencing

One-year-old *D. farinosus* materials potted in the growing chamber were used for RNA sequencing. The samples included branch, culm sheath, internode, lateral bud, young and mature leaves, node, root, sheath of bamboo shoot, and shoots of different height, which were collected for RNA sequencing to be performed. Each sample had three biological replicates. Total RNA was extracted by using TRIzol reagent (Tiangen, Beijing, China), and DNase I was used to purify potentially genomic DNA. The quantity and the quality of total RNA were checked by using a NanoDrop 2000 spectrophotometer (Thermo Fisher Scientific, Beijing, China). All 14 samples were subjected to the HiSeq 2500 platform (Illumina, Beijing, China), and the data of RNA sequencing (**Supplementary Table S3**) was used to construct the heat map of the *DfSUT* genes.

### Overexpression of *DfSUT4* in *N. tabacum*

The recombinant plasmid of pCAMBIA1303-N-DfSUT4 with CaMV 35S promoter was transferred into *Agrobacterium tumefaciens* EHA105. Transgenic tobacco overexpression lines were obtained by the leaf disc method (Zhang et al., 2019). Infected tobacco leaf discs were cultivated in the dark on MS medium containing 9 mg/L Hyg, 400 mg/L cephalexin, 0.5 mg/L 6-BA, and 0.1 mg/L NAA at 27 ± 1°C for 2 days. The regenerated shoots were transferred to 1/2 MS medium with 9 mg/L Hyg, 400 mg/L cephalexin, and 0.1 mg/L NAA for the formation of whole plants. Genomic DNA was extracted from the leaves of transgenic plants and wild-type and amplified under the following conditions: preheating at 95°C for 5 min, followed by 30 cycles of denaturation at 95°C for 30 s, annealing at 60°C for 30 s, extension at 72°C for 2 min, and finally extension at 72°C for 10 min. The PCR products were detected by 1% agarose gel electrophoresis to confirm the insertion of *DfSUT4* into the transgenic plants. Then, qRT-PCR was used to detect the expression of *DfSUT4* in transgenic plants. The internal reference gene was *N. tabacum NtActin* (Wang et al., 2022).

### GUS staining

Fresh plant organs of transgenic tobacco were fixed by 90% acetone on ice for 20 min and then placed in GUS staining solution containing 2 mM X-Gluc in GUS assay buffer (200 mM phosphate buffer, pH 7.0, 0.1% Triton X-100, 20 mM EDTA, and 0.5 mM potassium ferricyanide and potassium ferrocyanide), vacuum infiltrated, and incubated at 37°C overnight. The stained samples were then dehydrated in an ethanol series. Photographs were taken with a stereomicroscope.

## Data availability statement

The datasets presented in this study can be found in online repositories. The names of the repository/repositories and accession number(s) can be found below: https://www.ncbi.nlm.nih.gov/, PRJNA923443.

## Author contributions

SHu and YC planned and designed the research. BD, MZ, SC, SHa, and LW performed all experiments and analyzed the data. XG and BD wrote the original manuscript. SHu and YC proofread the manuscript. All authors contributed to the article and approved the submitted version.
